# Special Issue on Plant and Marine-Derived Natural Product Research in Drug Discovery: Strengths and Perspective

**DOI:** 10.3390/ph15101249

**Published:** 2022-10-11

**Authors:** Noélia Duarte

**Affiliations:** Research Institute for Medicines (iMED.Ulisboa), Faculdade de Farmácia, Universidade de Lisboa, Av. Prof. Gama Pinto, 1649-003 Lisboa, Portugal; mduarte@ff.ulisboa.pt

For centuries, nature has been an inspirational source for the discovery of traditional remedies and drugs used in modern medicine. Natural-based treatments continue to be employed for primary health care, particularly playing a significant role in folk medicine. In addition, we are currently observing an increasing use of natural-products-based supplements from botanical and marine sources, making their standardization and scientific validation a priority to guarantee the safety of these products. Natural products and/or synthetic derivatives using their novel structures have also been of utmost importance in drug discovery and development in several clinical areas. After a period of discredit and reduced investment, we are now witnessing a renewed interest from the pharmaceutical industry and scientific community due to the urgent need to develop new drugs.

This Special Issue of *Pharmaceuticals* has assembled eleven original and six review articles dedicated to plant and marine natural products research, contributing to the scientific validation of their use and opening new perspectives in drug discovery.

Yousefi-Manesh et al. [1] studied the effect of isofuranodiene, a furanosesquiterpene isolated from *Smyrnium olusatrum* essential oil, on the oxidative stress and inflammatory response in a rat model of ischemic stroke, by assessing several biochemical markers, pathology of the hippocampi cells, and behavioral assays. Pre-treatment with isofuranodiene decreased the levels of pro-inflammatory cytokines and the lipid peroxidation indicator malondialdehyde, as markers of neuroinflammation in ischemic stroke and oxidative stress, respectively. Moreover, the pre-treated animals showed improved behavioral activity after ischemic stroke, and a faster recovery was also observed. Despite the need for further pharmacokinetic and toxicological studies, isofuranodiene may be considered a lead compound for discovering new treatments for brain ischemia.

Searching for new therapies for central nervous system (CNS) tumors, Studzińska-Sroka et al. [2] screened the biological potential of acetonic extracts of the lichens *Parmelia sulcata*, *Evernia prunastri*, and *Cladonia uncialis*, and their major metabolites salazinic acid, evernic acid, and (−)-usnic acid. The extracts and pure compounds were evaluated for their cytotoxicity against A-172 and T98G cell lines, inhibition of kynurenine pathway enzymes, and anti-inflammatory, antioxidant, and anticholinergic activities. In addition, the penetration of salazinic acid, evernic acid, and (−)-usnic acid through the blood–brain barrier (BBB) was also determined. The authors suggested that (−)-usnic acid, with its ability to cross the BBB and reduce cell proliferation, can be considered a promising lead compound for glioblastoma multiforme treatment, one of the deadliest tumors of the CNS.

Garlic (*Allium sativum*) and onion (*Allium cepa*) are two edible plants widely consumed all over the world. In recent years, several biological actions have been attributed to different organosulfur products, such as thiosulfinates and thiosulfonates, obtained from these species. Sorlozano-Puerto et al. [3] evaluated the antibacterial and anti-candidiasis activity of propyl-propane thiosulfinate and propyl-propane thiosulfonate, two volatile compounds derived from *Allium cepa*. Moreover, the ability of the compounds in gaseous phase to inhibit bacterial and yeast growth was also assessed. Propyl-propane thiosulfonate was the most promising compound showing activity against different isolates of *Candida* spp. from human clinical samples, even at the gas phase, which, in the future, could be potentially useful in lung therapy.

*Posidonia oceanica* is a marine plant used in traditional medicine for the treatment of several health conditions, such as diabetes and inflammation. The aim of the study carried out by Vasarri et al. [4] was to analyze the ability of *P. oceanica* hydroalcoholic extract to trigger autophagy and reduce intracellular lipid accumulation, in an in vitro model of hepatic steatosis using the human hepatoma cell line HepG2. The authors found that the extract protected against lipid accumulation in HepG2 cells by promoting autophagy through inhibition of the mTOR pathway, offering new insights for possible complementary treatments of non-alcoholic fatty liver disease.

Ntungwe et al. [5] screened sixteen *Plectranthus* species acetone extracts for the in vitro antioxidant and antimicrobial activities against yeasts, and Gram-positive and Gram-negative bacteria. The *P. hadiensis* and *P. mutabilis* extracts showed the highest activity against *S. aureus* and *C. albicans*. Moreover, using the *Artemia salina* assay as a marker of general toxicity, *P. hadiensis* and *P.ciliatus* were selected and further tested for their cytotoxicity, showing low activities in human colon, breast, and lung cancer cell lines. 7α-acetoxy-6β-hydroxyroyleanone isolated from *P. hadiensis* was found to be twelve-fold more active than the extract in the triple-negative breast cancer cell line (MDA-MB-231S), being proposed as a promising lead compound for the development of new anti-cancer drugs.

The use of herbal compresses is widespread in traditional Thai therapies to relieve muscle pains, stress, and strains. Aiming at finding out the mechanisms of action and the bioactive constituents responsible for the anti-cellulite activity of a previous formulated herbal compress, Ngamdokmai et al. [6] presented, in their article, the preclinical effects of essential oils, extracts, and main monoterpenoid constituents on cellular lipid accumulation, triglyceride content, and vasodilatation in rat aortae. The authors concluded that the mixed oils have vasodilation activity, and all the tested samples were effective inhibitors of adipogenesis and lipolysis inducers, corroborating their application in cellulite treatment.

Melatonin was previously shown to exert a renoprotective effect in a rat model of diabesity-induced kidney injury. In their study, Aouichat et al. [7] further investigated whether melatonin could suppress the renal endoplasmic reticulum (ER) stress response and downstream unfolded protein response activation, shedding light on the beneficial effect of melatonin supplementation on ER stress-induced kidney damage under diabesity conditions.

*Asphodelus* L. species have been known for both food and therapeutic uses. Madia et [8] reported the isolation and identification of a polysaccharide (inulin-type fructan) from the alkaline extract of *Asphodelus ramosus* root tubers.

The goal of the study performed by Mota et al. [9] was the development of a topical ethosome-based formulation of fresh elderflower (*Sambucus nigra*) extract, to prevent its degradation and to obtain a slow release of bioactive compounds. The ethosomes were characterized in terms of their size and morphology, stability over time, entrapment capacity, and extract release profile. The extract-loaded ethosomes presented collagenase inhibition activity and very good results in the human skin compatibility assay using a semi-solid formulation.

Amphotericine B is a macrolide antibiotic clinically used to treat *Candida*, *Cryptoccocus*, and *Aspergillus* infections, and leishmaniasis. The intravenous administration of this antibiotic has severe side effects which have prompted the development of various formulations. The low solubility of amphotericin B also hinders its topical administration. In order to promote the diffusion of the drug through the skin in the treatment of cutaneous candidiasis, Espinoza et al. [10] developed a topical gel of amphotericin B and *Bursera graveolens* (palo santo) essential oil. The authors evaluated the physicochemical parameters, stability, in vitro release profile, and ex vivo permeation in human skin of the formulation, concluding that the topical gel of amphotericin B enriched with *B. graveolens* essential oil could be an alternative to the treatment of cutaneous candidiasis.

Consistent with the increasing use of nanotechnology for improving drug delivery, Adedokun et al. [11] compared the cytotoxic activity of *Hymenocardia acida* crude methanolic extract with the cytotoxicity of the extract loaded in PLGA nanoparticles, against human lung (H460), breast (MCF-7), and colon (HCT 116) cancer cell lines. *H. acida* extract showed to be cytotoxic against the lung cancer cell line and the solubility was improved through encapsulation. However, the encapsulated extract presented a decreased cytotoxic activity against all cell lines tested, a fact that could be due to the sustained delay in the release of the extract from the PLGA nanoparticles.

Mottaghipisheh et al. [12] reviewed the phytochemical and biological properties of the glycosylated flavone linarin (acacetin-7-O-rutinoside), which has been identified in several species of the Asteraceae, Lamiaceae, and Scrophulariaceae families. Several studies have reported promising in vitro and in vivo bioactivities, particularly on the central nervous system, arthritis, and osteoporosis disorders.

Several *Euphorbia* species have been used in traditional medicine, and have also been the source of a huge number of interesting bioactive compounds, including terpenois and flavonoids. Magozwi et al. [13] reviewed the phytochemistry, biological properties, and therapeutic potential of Euphorbia flavonoids, published in the literature from 2000 to 2020. Several bioactivities have been reported either for *Euphorbia* extracts or isolated flavonoids, particularly antiproliferative, antimicrobial, and antioxidant activities.

*Garcinia indica* is a species from the mangosteen family (Clusiaceae), with culinary, industrial, and therapeutic applications. The review by Lim et al. [14] highlighted recent studies regarding the in vitro and in vivo biological activities of this species, such as antioxidant, anti-inflammatory, anti-obesity, and hepato- and cardioprotective effects.

Over the last few decades, many studies have focused on the nutritional value and health-promoting properties of anthocyanins. Gonçalves [15] reviewed the chemical structure of these flavonoids, their main dietary sources, and bioavailability. They also pointed out the most recent results on the potential health benefits from the daily intake of anthocyanin-rich foods, as well as their possible pharmacological mechanisms of action.

Geraldes et al. [16] gathered and reviewed the literature on mycosporines and mycosporine-like aminoacids, summarizing their physicochemical features and biosynthesis, occurrence and distribution in nature, and phytochemical studies. They also reviewed their biological activities and biomedical and non-biomedical applications. Moreover, the authors constructed a chemical database available online for free at http://www.cena.usp.br/ernani-pinto-mycas (accessed on 14 September 2022).

Human African trypanosomiasis (sleeping sickness) and American trypanosomiasis (Chagas disease) are two protozoan neglected tropical diseases whose pharmacological therapies have serious limitations. Therefore, the discovery and development of new drugs are urgent, and natural products from plants are a promising approach to achieve this goal. In their comprehensive review, Durão et al. [17] gathered and discussed data published in the literature regarding terpenic compounds with antitrypanosomal activity against *T. brucei* and *T. cruzi*, covering the period from 2016 to 2021, and emphasizing the most promising bioactive terpenoids.

The 17 articles published in this Special Issue further strengthen the potential of natural product research for the discovery of new drugs and overall contribute to the scientific validation of their use.
